# Will virtual rehabilitation replace clinicians: a contemporary debate about technological versus human obsolescence

**DOI:** 10.1186/s12984-020-00769-0

**Published:** 2020-12-09

**Authors:** Tal Krasovsky, Anat V. Lubetzky, Philippe S. Archambault, W. Geoffrey Wright

**Affiliations:** 1 Department of Physical Therapy, University of Haifa, Haifa, Israel; 2 Pediatric Rehabilitation Department, Sheba Medical Center, Ramat Gan, Israel; 3 Department of Physical Therapy, Steinhardt School of Culture Education and Human Development, New York University, New York, NY USA; 4 School of Physical & Occupational Therapy, McGill University, Montreal, Canada; 5 CRIR - Centre de Recherche Interdisciplinaire en réadaptation, Montreal, Canada; 6 Neuromotor Sciences Program, Department of Health and Rehabilitation Sciences, Temple University, Philadelphia, PA USA

**Keywords:** Automation, Artificial intelligence, Technology, Futurism, Clinical roles, Virtual reality, Telehealth

## Abstract

This article is inspired by a pseudo Oxford-style debate, which was held in Tel Aviv University, Israel at the International Conference on Virtual Rehabilitation (ICVR) 2019, which is the official conference of the International Society for Virtual Rehabilitation. The debate, between two 2-person teams with a moderator, was organized by the ICVR Program committee to address the question “Will virtual rehabilitation replace clinicians?” It brought together five academics with technical, research, and/or clinical backgrounds—Gerry Fluet, Tal Krasovsky, Anat Lubetzky, Philippe Archambault, W. Geoffrey Wright—to debate the pros and cons of using virtual reality (VR) and related technologies to help assess, diagnose, treat, and track recovery, and more specifically investigate the likelihood that advanced technology will ultimately replace human clinicians. Both teams were assigned a side to defend, whether it represented their own viewpoint or not, and to take whatever positions necessary to make a persuasive argument and win the debate. In this paper we present a recapitulation of the arguments presented by both sides, and further include an in-depth consideration of the question. We attempt to judiciously lay out a number of arguments that fall along a spectrum from moderate to extreme; the most extreme and/or indefensible positions are presented for rhetorical and demonstrative purposes. Although there may not be a clear answer today, this paper raises questions which are related to the basic nature of the rehabilitation profession, and to the current and potential role of technology within it.

## Background

### Definition of the problem

To debate the question “Will virtual rehabilitation replace clinicians?” it is necessary to provide a definition for *virtual rehabilitation*. But first, we must define virtual reality (VR). Jaron Lanier, who is credited with first coining the term *virtual reality*, has defined it in many ways, some more poetic than others [1], but it can be succinctly expressed as “an artificial environment which is experienced through sensory stimuli (such as sights and sounds) provided by a computer and in which one's actions partially determine what happens in the environment” [2]. We also provide an accepted definition of *rehabilitation*, to help better define the term virtual rehabilitation. Rehabilitation services help an individual maintain, restore, or improve skills and function for daily living that have been lost or impaired because a person was sick, hurt or living with temporary or permanent disability [3]. This leads to the definition for “virtual rehabilitation”, which is a neologism formed by combining the two terms just defined. *Virtual rehabilitation* refers to use of applications either based on, or improved by, VR [4] to support or enhance human health and function [5]. Although different views exist (e.g. [6]), for this discussion we chose to investigate the topic using a broad perspective, where VR may be a component of a complex system which incorporates additional physical interfaces for interaction with a patient—such as physical assistance (robotics) or augmented sensory feedback (haptics) [7]. The use of haptic feedback may be less developed and therefore less frequently included than the other types of VR sensory feedback (e.g. visual and auditory feedback), but haptics can help increase immersion and perceived realism in virtual environments when performing sensorimotor tasks [7]. We use this more inclusive definition for the term *virtual rehabilitation* in order to facilitate the discussion of the question, i.e., what type of virtual rehabilitation systems would be required to replace clinicians.

Almost 20 years ago, in a keynote address in the 1st International Workshop on VR Rehabilitation, it was noted by Dr. Grigore Burdea that “Certain unwise (and short-sighted) technologists have proclaimed that VR will replace the therapists altogether with computers” [4]. In the 20 years since then this has not happened, but the topic remains thought-provoking, and raises important questions related to the basic nature of the rehabilitation profession, and the current and potential role of technology within it. Furthermore, recent technological developments may (or may not) change this outlook in the future. We do not hope to resolve the debate in this paper, but instead to make a contemporary commentary on an insidious conflict that Karl Marx wrote about nearly two centuries ago: “The instrument of labour, when it takes the form of a machine, immediately becomes a competitor of the workman himself.” [8].

## The debate: will virtual rehabilitation replace clinicians?

### The “yes” side

The concept of virtual rehabilitation replacing clinicians carries with it an unavoidable negative sentiment among practicing clinicians and researchers in this field, a concern that is not unique to rehabilitation professionals. The advances of artificial intelligence (AI) in the last few decades have led futurist Ray Kurzweil to suggest that we are at the “knee” of an exponential curve in terms of technological development [9], and that the effects of these developments in the next few decades will be radical in terms of merging physical and virtual reality. Technological advancements in the years to come will inevitably change the job market. Ford [10] identified the current advances of information technology as a tipping point which will change the face of the job market in different fields in unpredictable ways. Not only will the low-skilled workforce be replaced by technology, but also highly skilled professions which require both intellectual aptitude and years of training, are expected to undergo dramatic changes. These changes include, in some cases, a new division of labor (by “offshoring” parts of the manufacturing process to other countries using technology to maintain service quality) and in other cases replacement of human labor by robots or computers.

Virtual rehabilitation may be particularly well-suited to replace humans in the upcoming "era of the machines". In recent years, a massive surge of VR applications has been used for motor rehabilitation of the upper limb [11], posture and gait [12, 13] as well as neuropsychological interventions [14, 15]. Enthusiasm regarding VR as a rehabilitation tool stems from several sources. First, VR is fun and enjoyable for most people, and a high degree of motivation assists with adherence to interventions [16]. Second, by using computerized assessment of performance in VR, clinicians can keep track of quantifiable indices of performance over time which enables optimal selection of difficulty levels for individual patients as well as optimized goal setting within the system [17]. Thus, therapists can more easily track performance and learning. Importantly, a VR rehabilitation session can be delivered remotely while the patient is at home; telerehabilitation programs are showing promise by obtaining comparable results to therapist-supervised programs e.g. for people after stroke [18, 19] and people with Parkinson's disease [20, 21] at lower costs [22]. VR rehabilitation in the form of telerehabilitation can be provided on a large scale and for longer durations. This is particularly important because life expectancy continues to increase: average global life expectancy increased by 5.5 years between 2000 and 2016, the fastest increase since the 1960s [23]. Home rehabilitation services help aging adults improve or maintain their quality of life, physical function and independence; in doing so it extends their time in the community and away from hospitals [24, 25]. Despite this evidence, many clients who could benefit from home rehabilitation services do not receive them [26] and virtual rehabilitation can play a key role in this solution. The advantages of virtual rehabilitation discussed here, including cost-effectiveness and provision of care to remote areas not accessible to standard care, can increase health care quality and availability for all and support healthy aging in place.

The advantages of VR may imply that application of this technology should be widespread and that clinicians should, indeed, start “fearing for their jobs”. However, this is currently not the case. In fact, the health care system, in general, is traditionally considered less vulnerable to the type of change advocated by Ford [10]. Health care professionals, including those in rehabilitation, may be more indispensable than other workers. In the widely-cited paper of Frey & Osborne [27], the authors ranked 702 professions according to their risk of computerization. Physical therapists were ranked in the top 15% (90 out of 702) for resistance to automation (smaller numbers denote a lower chance of computerization) and occupational therapists were even more “safely” ranked (6 out of 702, top 1%), suggesting that the risk of unemployment for rehabilitation professionals is still low. Indeed, important barriers exist for the integration of virtual rehabilitation into clinical practice [28]. These barriers include aspects related to the technology itself (which may not meet the therapists’ and clients’ needs), the infrastructure (allowing time and technical support for technology integration) and the therapists (who do not feel competent enough with the technology). Overcoming these barriers may seem daunting, but other fields have proven that technology is increasingly accomplishing feats which were previously thought to be impossible. For example, in “The New Division of Labor: How Computers are Creating the Next Job Market”, authors Levy and Murnane [29] stated: “…executing a left turn against oncoming traffic involves so many factors that it is hard to imagine discovering the set of rules that can replicate a driver’s behavior" (p. 20), suggesting that the acquisition of tacit knowledge is impossible for machines. However, only 15 years later, "deep learning" has revolutionized the field of autonomous vehicles [30] to a point that autonomous vehicles are now performing test drives in the United States [31]. In fact, deep learning algorithms are increasingly performing tasks in various fields which were previously considered impossible (from music to poetry). AI has already made its value known in the medical fields related to cancer detection, heart disease, stroke recovery, and for programming human–machine interfaces to help recover movement control following spinal cord injury [32]. Extrapolating to the proliferation of virtual rehabilitation into clinical use, we suggest that near future advancements of technology can change the field of rehabilitation in fundamental ways.

It is thus suggested here, that the question “will virtual rehabilitation replace clinicians?” may be phrased better as “*when* will virtual rehabilitation replace clinicians?”. Although the answer to this question is speculative, one can highlight the ways to overcome existing barriers which inhibit an early disruption of the rehabilitation field by technological advancement. These can be divided into three main domains: (1) the technology, (2) the clinicians, and (3) the patients. Examining the first domain of technological progression, it is proposed that substantial improvements are needed in terms of *ease of use* (e.g. set-up time is an important barrier for VR implementation [28, 33]), *reliability* of the technology (e.g. works every time such that the clinician and patient develop trust in the system) and *capabilities.* New capabilities of VR systems can be, for example, the addition of sensors, which can potentially measure any physiological and emotional parameter [34]. Some of these capabilities are already being implemented. For example, cognitive load can be evaluated via direct assessment of brain activation, using functional near-infrared spectroscopy [35] or electroencephalography [36]. When integrated into virtual reality exposure therapy sessions [37], a combination of VR-based exposure and close monitoring of patient cognitive state improved both physiological and cognitive symptoms of anxiety. This type of technology, which until recently was considered too costly to be clinically applicable, is becoming a consumer product, and simple low-cost devices already exist on the market, for example to assess the level of relaxation/arousal through electroencephalography [38]. When combined with VR systems, close monitoring of physiological and sensory states can improve algorithms for goal-setting during practice, by generating adequate challenge and avoiding patient frustration. The accurate quantification of patient performance and the complexity of machine learning allow automated or semi-automated goal setting, which has the potential to further reduce treatment costs, maintain patient engagement and effectively lead patients to achieve treatment goals [17]. In some fields, such as post-stroke rehabilitation, automated and semi-automated goal setting in VR is showing promise in improving patient outcomes [39, 40]. The second domain, that of *the clinicians*, may be addressed via educational strategies that help train future clinicians for a more technological working environment and effectively prepare them for new and different professional roles. A recent study demonstrated that a virtual rehabilitation therapy program is equally effective when supervised by a physical therapist or by a rehabilitation assistant [41]. These results should raise a red flag for educators of rehabilitation professionals, suggesting that in a future working environment, a clinician needs to assume new roles and responsibilities and let go of some of their traditional roles in order to survive. Embracing the challenges of a new work environment can lead clinicians to focus on aspects which were not previously considered to be a main part of their role—or to assume new roles altogether. In this environment, where the technology is more advanced and the clinicians are ready to assume different professional roles, the third and perhaps most important domain, that of the *patients* themselves, can also evolve. Patients today may be “technophobic” and apprehensive towards virtual rehabilitation applications, but effective technology and a positive approach by a clinician can ameliorate their view of virtual rehabilitation. Additionally, technological advancements in AI have begun to allow for rapid adaptation of the therapy to the current needs of the patient. Such personalization of the therapy in real-time during their therapeutic exercises will not only improve outcomes as mentioned above [29, 30], but it can help the patient achieve an optimal state of experience, i.e. “flow” [42]. Achieving such a state of increased attentional engagement, cognitive absorption, and mental arousal can tap into a patient’s intrinsic motivators [43], which can make therapy more appetitive than aversive.

To summarize the “yes” side, given the incredible advancement of technologies in recent years, we can safely state that acceptance of VR-based tools in the clinic is already happening and with that a pathway towards automation exists, which has been witnessed in human history many times before. Calls for change in content of the professional practice in light of advancing technology are being raised in other professions such as nursing [44], and while the timeframe for this change is not provided, it is suggested here that it may be sooner than we think.

### The “no” side

Rapidly evolving technologies are constantly adding tools to rehabilitation that were not available in the recent past. Clinicians can now immerse their patients in different virtual worlds to reduce pain or anxiety, they can encourage them to move by playing games, they can quantify performance measures that are not easily detectable by the naked eye. However, although automatization has happened in many fields, the field of rehabilitation does not seem to follow the same rate of technological change [10]. The following are several arguments as to why we cannot take clinicians out of this equation and even if it were possible, whether it would be advisable.

If virtual rehabilitation were ever to replace physical rehabilitation a required first step would be to know exactly what it is that we are replacing. Physical rehabilitation can broadly be defined as the process of restoring and regaining physical strength and function [45]. The process often involves contact with various health disciplines, such as physiatrists, physical, occupational, speech and recreation therapists, psychologists, and nurses. Input from some or all of these professionals is typically required to help an individual achieve the highest level of functional independence and quality of life. Replacing physical or cognitive rehabilitation would require clear definition of action plans according to ‘standard of care’ and training software according to a finite number of clinical decision options. And yet defining ‘standard of care’ has often proven to be challenging. We suggest that a main reason for this is the high degree of personalization of the treatment regimen which is required for planning and carrying out rehabilitation interventions. The International Classification of Functioning, Disability and Health [46] calls for accounting for personal and environmental factors as much as one should consider the pathology and impairments associated with a condition. The physician Sir William Osler stated: ‘it is much more important to know what sort of patient has a disease than what sort of disease a patient has’ [47]. With that, the hallmark of rehabilitation is the ability to individualize a program. As stated in Locsin and Ito, who asked whether robots can replace nurses: “knowing persons more fully as participants in their care is acclaimed best, rather than considering them as objects and recipients of care” (p. 5) [44]. Indeed, the act of providing a clinical rehabilitation service consists of much more than providing motor or cognitive exercises. The clinician, broadly, establishes a therapeutic alliance with each client. The confidence that clients have towards their therapists helps establish a collaborative approach. Therapeutic alliance increases clients’ commitment to their therapy, their satisfaction with interventions, and is directly linked to positive rehabilitation outcomes [48–50]. Rehabilitation professionals, as with any other healthcare professional, also have the duty of offering other services to their clients, above and beyond therapy for specific health issues. These roles include, among others, education to inform the clients, their family, and/or their caregivers about health conditions (etiology, symptoms, prevention measures, etc.). This needs to be proactively initiated by the therapist according to patient- and family-specific context (e.g. education, religion, mentality, relationships) and be done with maximal sensitivity. When needed, clinicians also refer clients to other members of the interprofessional team. If VR is to replace clinicians, it would have to do so not only to provide therapy to clients based on their specific needs, but also replace clinicians in their other roles—a feat which is still far from possible in our current technological state of affairs.

Proponents of technology claim that virtual rehabilitation can increase accessibility to care due to reduced costs and removal of barriers due to geographical distance, and thus promote equality. However, access to technology, even at the basic levels of running a computerized application, would require reading and following instructions, solving basic technological malfunctions (e.g. faulty internet connection) and thus would require a minimal level of physical and cognitive function, or the close assistance of a caregiver [51]. Furthermore, there exists a huge mismatch between the pace at which technology evolves and the pace of generating new evidence and implementing this evidence into clinical practice. The rapid pace of technology advancement inevitably generates an inability of clinicians to keep up with software and hardware versions [28]. If virtual rehabilitation were to replace all clinicians, who would develop the training modules? Who will assure knowledge transfer and skill development? Here, proponents of technology would argue that AI will assume this role. However, it appears that we are still far from that scenario. While there have been tremendous advances in AI applications over the past years, current algorithms may oversimplify their classification process, to a point that safety may be of concern. For example, researchers have shown that by adding coherent noise to an image, they can trick image-recognition algorithms such that these fail at an alarming rate [52]. Likewise, the Tencent Keen Security Lab was recently able to trick a Tesla car to falsely recognize a stop sign as a speed limit sign, using specifically designed stickers placed on the sign [53]. Much development is still required in terms of AI algorithms before these can be considered as safe and reliable enough to remove the human in the loop by taking the clinician out of the process.

An additional concern for virtual rehabilitation is its lack of flexibility. To this end, the quality of the VR experience, as well as the accuracy of tracking and interpretation of movements, depend on the interaction between human movement, a computer program and an interface, e.g. a controller, a camera, a head-mounted display (HMDs) or a robotic device. Although considerable advances have been made in this respect (e.g. [54, 55]), VR rehabilitation applications are rarely independent of a specific device (e.g. HMD, glove, camera) and the efforts to migrate a VR application from one platform to another are costly [51]. The lack of flexibility of VR applications is demonstrated also when a modification to a VR session is required in order to fit a specific patient’s needs. Although the ability to flexibly modify training parameters is an asset of VR [56], the conflict between overwhelming the therapist with “too many controls” and providing a “one size fits all” solution is still an issue today [28]. We suggest that the fact that the industry has been unable to solve this problem in more than 15 years of research [51] stems from a fundamental issue with the compatibility of virtual rehabilitation with the requirements of the clinical world. Balancing flexibility with ease-of-operation to support effective training may be an insoluble problem, which would make it a limiting factor keeping VR from replacing clinicians.

A final but alarming point is safety: what happens when things go wrong? HMDs, for example, have the advantage of providing 3D, stereoscopic vision, increasing realism and sense of presence. However, a still unsolved issue is the possibility of appearance of symptoms of cybersickness, such as nausea, vertigo and disorientation [57]. Although these side-effects vary by device, recent research demonstrates that across platforms, these symptoms may increase with exposure time [58]. Effects of using a VR device may vary by task, as well. Indeed, walking on a treadmill while viewing a congruent scene through an HMD is associated with greater postural instability, as well as some changes in gait patterns, as compared to walking without an HMD [59]. This suggests that some tasks may not be fully transferrable to a VR environment, and highlights the importance of a clinician in choosing the proper VR rehabilitation application and supervising their performance. Finally, if an emergency occurs which is associated with technology—and this may occur even in healthy users of technology (e.g. [60]), the question of responsibility of the health care provider, VR company or supervising therapist inevitably arises, and this question is far from settled.

To summarize the “no” side, despite the incredible advancement of technologies in recent years, we can safely state that “technologies are only as good as their makers” [61]. The issues with which the virtual rehabilitation community was dealing with more than a decade ago are still relevant today. The question of taking clinicians out of the equation involves a great leap in abilities of technology, which has not occurred over decades of research and application of VR technology in rehabilitation. Even if this change were to happen, it may lead to deterioration in the level of care, to social inequality, and to reduced patient safety. These are unacceptable risks for the rehabilitation field.

## Conclusion

**Fig. 1:**
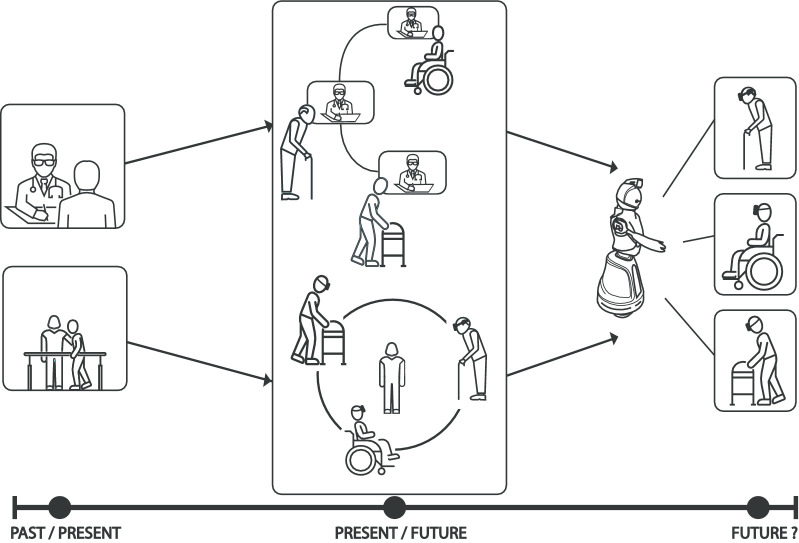
The changing roles of clinicians and technology in rehabilitation. Advanced technology including virtual reality, telerehabilitation, robotics, as well as sophisticated machine learning algorithms are transforming traditional clinical care. The clinician’s role in the past and present has been critical to patient care. While this still remains true, changing roles of the clinician include when a remote (telehealth) or onsite clinician can treat one or more patients with the aid of technology that has increased reach, reduced clinician burden, and/or automated assessment and intervention. In the future there are many ways that the clinician may continue to serve the patient population, however, one possible future being debated is whether advanced technology could ever completely replace the clinician

Technology has been advancing at an exponential rate for many decades (Moore’s Law), hence where we will be in 10–20 years in not yet known. For the rehabilitation field, it is unknown whether we will have a contemporary Luddite rebellion in our future or instead an age of technophilia, which will allow for a rapid adoption of virtual rehabilitation. The points raised in this debate highlight the complexity of the issues surrounding this question. Prior to the debate, 100% of the clinicians, scientists, and technologists in the audience all voted ‘no’. However, a post-debate vote revealed the audience was split in their support for either side. We believe that this is due to the fact that while the current state of affairs clearly supports a “no” (as evidenced by the fact that VR has not replaced clinicians), considering the possibilities, the future state of affairs may suggest a “maybe” (Fig. 1). An example of this can be drawn from recent events surrounding a pandemic that urgently increased the need for telemedicine in order to facilitate treatment delivery in remote areas and reduce disease transmission in densely populated areas. The level of need rose so abruptly that some governments passed policies restructuring how telemedicine could be billed. In the United States, insurance companies made it possible to provide acute care to patients using a combination of telehealth and on-site clinicians [62]. While circumstances surrounding this are extreme, they highlight a compromise that should be considered. Clinicians and technologists should work together with a strong consideration for how environmental, governmental, and market forces may help or hinder adoption of the best evidence-based practices.

In the process of technological change, humans *are* the driving force [61]. Clinical expertise, based on years of research and experience, needs to be an important input in the generation of novel VR solutions such that these will produce meaningful and effective experiences for patients. Furthermore, novel VR rehabilitation applications may not necessarily replace every aspect of the clinician’s role. Instead, clinicians should be encouraged to acknowledge the advantages offered by technology, which may free them of some aspects of the profession and allow development in others. The reality is that while VR may not replace clinicians, under certain circumstances—which involve better technology and increased acceptance from all stakeholders (namely patients, caregivers, clinicians, healthcare and insurance providers)—it may replace some aspects of a clinician’s current job description in upcoming years. Clinicians, like people from other professions, will need to perform in an eco-system where technology is a key player. This will necessarily involve some adaptation in the thought process and decision making, which we are all currently going through. Rehabilitation specialists may benefit from exposure to technology early in their training, which will make them more ready to adopt new tools and expand their toolbox. For technologists, it is clear that any progress should be made in close collaboration with clinicians, patients, caregivers, and healthcare and insurance providers so as to increase availability and usability of the technology. It is essential that technological solutions for rehabilitation are trustworthy in order for them to be useful. What history may tell us, is that careful consideration of these issues and measured progress will allow all stakeholders to be involved in the advancement of the clinical approach to care, which will best serve all involved parties, but first and foremost the patients.

## Data Availability

Not applicable.

## Abbreviations

AI: Artificial intelligence
fNIRS: Functional near-infrared spectroscopy
HMD: Head-mounted display
ICVR: International Conference on Virtual Rehabilitation
VR: Virtual reality
